# Zika virus infects human blood mononuclear cells

**DOI:** 10.1186/s12879-019-4622-y

**Published:** 2019-11-21

**Authors:** Carolina V. Messias, Julia P. Lemos, Daniela P. Cunha, Zilton Vasconcelos, Lidiane M. S. Raphael, Myrna C. Bonaldo, Bruno Cister-Alves, Dumith Chequer Bou-Habib, Vinicius Cotta-de-Almeida, Wilson Savino, Daniella A. Mendes-da-Cruz

**Affiliations:** 1 Laboratory on Thymus Research, Oswaldo Cruz Institute, Oswaldo Cruz Foundation, Ave. Brasil 4365, Rio de Janeiro, 21045-900 Brazil; 2 National Institute of Science and Technology on Neuroimmunomodulation, Oswaldo Cruz Institute, Oswaldo Cruz Foundation, Rio de Janeiro, Brazil; 3 High complexity Laboratory, Fernandes Figueira National Institute of Health in Mother, Infant and Adolescent, Oswaldo Cruz Foundation, Rio de Janeiro, Brazil; 4 Laboratory of Molecular Biology of Flavivirus, Oswaldo Cruz Institute, Oswaldo Cruz Foundation, Rio de Janeiro, Brazil

**Keywords:** Zika virus, Lymphocytes, Monocytes, Flow cytometry, RT-qPCR

## Abstract

**Background:**

Zika virus (ZIKV) infection gained public health concern after the 2015 outbreak in Brazil, when microcephaly rates increased in babies born from infected mothers. It was demonstrated that ZIKV causes a congenital Zika virus syndrome, including various alterations in the development of the central nervous system. Although the infection of cells from the nervous system has been well documented, less is known in respect of ZIKV ability to infect immune cells. Herein, we investigated if peripheral blood mononuclear cells (PBMCs), freshly-isolated from healthy donors, could be infected by ZIKV.

**Methods:**

PBMCs from healthy donors were isolated and cultured in medium with ZIKV strain Rio-U1 (MOI = 0.1). Infection was analyzed by RT-qPCR and flow cytometry.

**Results:**

We detected the ZIKV RNA in PBMCs from all donors by RT-qPCR analysis. The detection of viral antigens by flow cytometry revealed that PBMC from more than 50% the donors were infected by ZIKV, with CD3^+^CD4^+^ T cells, CD3^−^CD19^+^ B cells and CD3^+^CD8^+^ T cells being, respectively, the most frequently infected subpopulations, followed by CD14^+^ monocytes. Additionally, we observed high variability in PBMC infection rates among different donors, either by numbers or type infected cells.

**Conclusions:**

These findings raise the hypothesis that PBMCs can act as a reservoir of the virus, which may facilitate viral dissemination to different organs, including immune-privileged sites.

## Background

Zika virus (ZIKV) is as an arthropod-borne virus of the *Flaviviridae* family, *Flavivirus* genus, which is divided in two phylogenetic lineages named Asian and African [1]. It is a single-stranded RNA virus protected by a capsid, which is covered by a lipid envelope. The genome encodes a single polypeptide that is cleaved into three structural proteins, capsid (C), pre-membrane (prM) and Envelope (E) proteins, and seven non-structural proteins (NS1, NS2A, NS2B, NS3, NS4A, NS4B and NS5). Structural proteins form the viral particles while non-structural proteins are involved with genome replication and viral particle assembly [2].

Cases of ZIKV infection remained rare and sporadic until 2007, when an outbreak occurred in the Yap Island in Micronesia [3]. Indeed, ZIKV infection gained national and global public health concern after an outbreak in Brazil, in 2015, when microcephaly rates increased in babies born from infected mothers [4]. A study performed in Slovenia revealed that after elective termination of pregnancy, ZIKV viral particles were detected in brain tissues of the fetus diagnosed with microcephaly [5] and ZIKV RNA was also identified in the amniotic fluid of pregnant women who delivered microcephalic babies [4]. Besides microcephaly, other neurological abnormalities were detected in babies born to infected mothers including cerebral calcifications, cerebellar dysplasia, ocular lesions, hearing loss and arthrogryposis [6]. Because ZIKV infection correlated with several neurological abnormalities during fetal development, the concept of congenital ZIKV syndrome has been proposed.

In adults, ZIKV infection is associated to Guillain-Barré syndrome [6, 7]. By March 2017, 31 countries and territories have reported cases of microcephaly and other central nervous system abnormalities possibly related to ZIKV infection during pregnancy, and 23 countries and territories have reported increased incidence of Guillain-Barré syndrome [8].

It is important to note that, after ZIKV spread, new routes of viral transmission were reported such as perinatal [9] and sexual transmission [10], and potential transmission through blood transfusion was suggested [11]. Additionally, active viral particles were detected in urine and saliva of infected patients [12], although no evidence of ZIKV transmission through contact with these fluids has been reported.

So far, little is known regarding the infection of immune cells and lymphoid organs by ZIKV. Persistent detection of ZIKV RNA copies was detected in lymph nodes of *Rhesus* monkeys for up to 72 days. The presence of the virus was confirmed in both paracortex and germinal centers of these lymph nodes by immunohistochemistry and in situ hybridization [13, 14]. Also, ZIKV genome was detected in macrophages, dendritic cells and B-cells of spleen and axillary lymph nodes of Rhesus monkeys. Noteworthy, the study described the presence of ZIKV RNA in T cells only in axillary lymph nodes from one animal [14]. Infection of PBMCs has been documented, although the results vary in terms of which cell types were actually infected (Foo et al. 2017; Michlmayr et al. 2017). It is possible that such differences are due to different viral strain used. In this work, using a Brazilian ZIKV, isolated from a human case, we investigated whether PBMCs from healthy donors are targets of ZIKV, and which subpopulations are in fact affected by this virus.

## Methods

### Zika virus and PBMC isolation and infection

ZIKV strain Rio-U1 used in the present study was isolated from the urine of a patient in Rio de Janeiro, Brazil in 2016 (GenBank Accession number: KU926309), as described elsewhere [12]. ZIKV was used to infect PBMCs after two passages in Vero cells.

Blood samples from 16 healthy donors were provided by the Hemotherapy Service of the Hospital Clementino Fraga Filho (Federal University of Rio de Janeiro, Brazil). PBMCs were obtained from buffy-coats preparations after density gradient centrifugation (Ficoll-Paque Premium 1.077; GE Healthcare Biosciences, PA, USA), and infected with 0.1 multiplicity of infection (MOI) for 2 h at 37 **°**C in an atmosphere containing 5% of CO_2_. Overall, 2 × 10^6^ PBMCs were infected and during infection, they were gently homogenized every 30 min. Cells were then washed, plated in 96 well plates (2 × 10^5^ cell/well) and maintained for 72 h at 37 **°**C in an atmosphere containing 5% of CO_2_. After PBMC isolation, all steps were performed in RPMI-1640 (Sigma-Aldrich, St. Louis, MO, USA), pH 7.2–7.5, supplemented with 10% fetal bovine serum (FBS) (Gibco, Thermo Fisher Scientific, Rockford, IL, USA), 2 g/L sodium bicarbonate (Sigma-Aldrich), 2 g/L HEPES (Invitrogen), and 1X antimycotic solution (A5955 - Sigma-Aldrich) at 37 **°**C in an atmosphere containing 5% of CO_2_.

As a control for some assays, ZIKV was inactivated for 2 h at 60 **°**C. Vero cells were infected with 0.1 multiplicity of infection (MOI) for 1 h at 37 **°**C in an atmosphere containing 5% of CO_2_. Medium containing viral particles was removed and fresh medium was added to the culture, which was incubated for 72 h. All steps were performed with RPMI-1640 supplemented as described above.

### Quantitative real time polymerase chain reaction (RT-qPCR) and viral load determination

Supernatants from PBMCs, infected or not with ZIKV, were harvested and the presence of ZIKV, Dengue virus (DENV) and Chikungunya virus (CHIKV) RNA particles was verified by RT-qPCR, using the Trioplex Real-Time RT-PCR Assay (Centers for Disease Control and Prevention, Atlanta, USA) according to the manufacturer’s instructions on BD MAX System (Becton Dickinson, San Jose, USA). Additionally, ZIKV active infectious loads were assessed using a standard curve starting from 1 to 1 million of plaque forming units per milliliter (PFU/mL) defined in VERO cells.

### Viral multiplication

To verify the production of new and active viral particles in PBMCs, we performed a titration of ZIKV in culture supernatants after infection. An aliquot of supernatant was harvested every 24 h after infection for 3 days and frozen at − 80 °C. Briefly, Vero cells were seeded in a 24-well plate (5 × 10^4^ cells/cm^2^), 24 h before inoculation. Serial dilutions of the supernatant were used to infect cell monolayers. After 1 h incubation at 37 °C, the supernatant was replaced by 2.4% CMC (carboxymethyl cellulose) in Earle’s 199 complete medium supplemented with 5% FBS, followed by incubation for 7 days at 37 **°**C. Cells were fixed in 10% formaldehyde, washed, and stained with 0.4% crystal violet. Viral titers were determined from the numbers of plaques visualized.

### Antibodies

PE-Cy7 mouse anti-human CD3 (Clone UCHT1), APC mouse anti-human CD4 (Clone RPA-T4), FITC mouse anti-human CD14 (Clone MφP9), FITC mouse anti-human CD19 (Clone HIB19), PECY5 mouse anti-human CD11c (Clone B-ly6), APC mouse IgG2b isotype control (Clone 27–35), APC-Cy7 mouse IgG2a isotype Control (Clone G155–178), FITC mouse IgG2b isotype control (Clone MPC-11) and purified mouse IgG1 isotype control (Clone MOPC-31C) were purchased from BD (San Jose, CA, USA), whereas APC/CY7 mouse anti-human CD8 (clone RFT8) was from Southern Biothech (Birmingham, AL, USA), PECy7 mouse IgG1 isotype control and goat anti-mouse IgG Alexa Fluor 546 were from Invitrogen (Invitrogen, Thermo Fisher Scientific, Rockford, IL, USA), PECY5 IgG2a isotype Control (Clone eBM2a) was from eBioscience (eBioscience, Thermo Fisher Scientific), and mouse anti-flavivirus (clone 4G2) monoclonal antibody was kindly provided by Biomanguinhos (Fiocruz, Rio de Janeiro, Brazil). The 4G2 antibody recognizes a conserved epitope in the envelope protein from flaviviruses, including ZIKV [15].

### Flow cytometry

PBMCs were trypsinized for recovery of adhered monocytes and subsequently stained with Foxp3 / Transcription Factor Staining Buffer Set (Invitrogen). Cells were incubated with fixation and permeabilization buffer for 40 min at 4 °C, followed by incubation with human serum for 20 min at 4 °C for Fc receptor blockage. Cells were then incubated with the 4G2 antibody for 1 h at 4 °C followed by incubation with Alexa 546 labeled goat anti-mouse antibody for 30 min. Between incubations, cells were washed with the permeabilization buffer. Subsequently, the labeling of membrane antigens (CD3, CD4, CD8, CD11c, CD14 and CD19) was performed for 30 min at 4 °C. Cells were then washed with PBS, fixed with formaldehyde 2% and analyzed by flow cytometry in a FACSCanto II device (BD Bioscience). Flow cytometry data analysis was performed through BD FACSDiva Software (BD Bioscience). The flow cytometry-based gate strategy adopted for these analyzes is shown in Additional file 1: Figure S1.

## Results

**Fig. 1:**
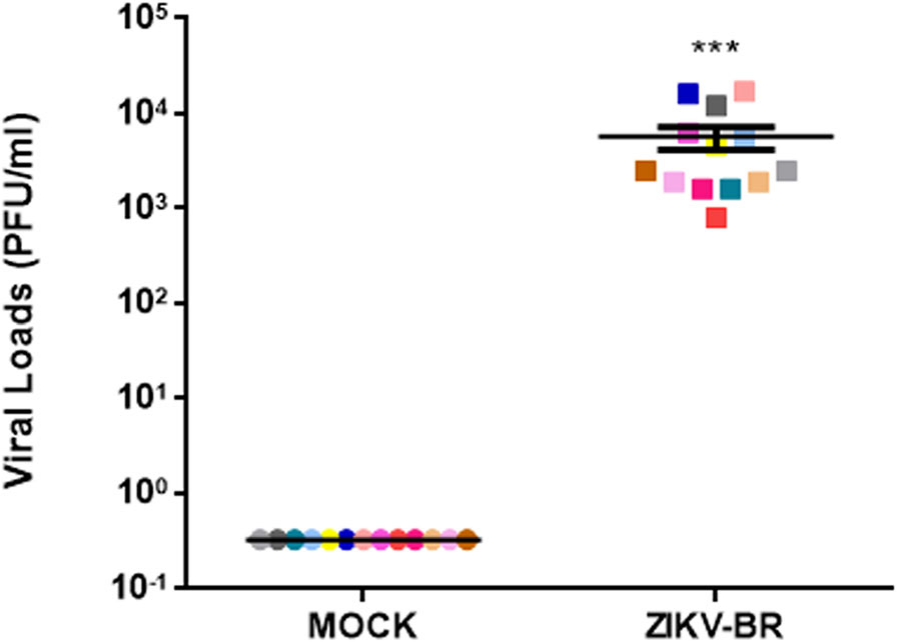
ZIKV virus infects peripheral blood mononuclear cells. PBMCs were infected by ZIKV at MOI 0.1 for 2 h and 72 h post-infection the supernatants were harvested and the presence of viral genome was verified by RT-qPCR. Viral loads were determined by absolute quantification based on a standard curve also performed by RT-qPCR and are represented as plaque forming units per milliliter (PFU/mL). Dots represent ZIKV viral loads in the supernatants of PBMCs (*n* = 13). Statistical analysis was performed with Wilcoxon matched-pairs test (***p < 0.001)

We first verified the ability of ZIKV to infect PBMCs under ex vivo conditions. At 72 h post infection, supernatants from PBMCs were harvested for determining viral proliferation through quantification of viral RNA genome copies. In all thirteen samples analyzed, we found high viral load levels in supernatants from PBMC cultures, ranging from 799.2 to 16,948.2 PFU/mL with an average of 5665.2 PFU/mL (Fig. 1; Table 1). Some donors presented viral loads higher than 10,000 PFU/mL (D6, D7 and D14), while only one showed less than 1000 PFU/mL (D5). Once we used Trioplex assay in all molecular tests, we can assume that none of the donors were positive for DENV or CHIKV during ZIKV inoculation assays.

**Table 1:** ZIKV RNA in supernatants of human peripheral blood mononuclear cells after 72 h of ex-vivo infection^a^

Donor	ZIKV viral load (PFU equivalent/mL)
D4	1589.0
D5	799.2
D6	15,700.4
D7	16,948.2
D8	6280.5
D9	4627.6
D10	5818.8
D11	1851.1
D12	1589.0
D13	1851.1
D14	11,568.3
D15	2512.3
D16	2512.3

^a^Table shows viral load (PFU/mL) in culture supernatants of ex-vivo ZIKV-infected human PBMCs from each healthy donor. PFUs were determined by absolute quantification performed by RT-qPCR. ZIKV viral loads for MOCK samples were always below 0.3 PFU/mL

To verify if the detected viral loads were due to active PBMC infections and not to passive uptake of viral particles, we performed viral titration in culture supernatants. At 24, 48 and 72 h post infection, supernatants from MOCK, inactivated ZIKV or ZIKV infected PBMCs were harvested and used to infect Vero cell monolayers. Our results demonstrated an increase in viral titrations over time, indicating active PBMC infections. We were not able to detect viral particles in the supernatant of MOCK or inactivated ZIKV infected samples (Additional file 2: Figure S2A). This result also reveals that the viral particles produced by PBMCs can infect other cells, since titration was performed in Vero cells (Additional file 2: Figure S2B). Overall, our data clearly show that, at least in the experimental conditions applied in this work, human PBMCs are targets for ZIKV.

To confirm the PBMC infection by ZIKV, we attempted to detect flavivirus envelope protein in infected cells by multicolor flow cytometry using the anti-flavivirus 4G2 antibody. We identified 4G2^+^ cells in 9 out of 16 samples - i.e., 56.25% (Table 2).

**Table 2:** Flow cytometry-based detection (%) of flavivirus envelope protein in subpopulations of human peripheral blood mononuclear cells after ex-vivo infection^a^

Donors	PBMC subpopulations (%)			
	CD3^+^CD4^+^4G2^+^	CD3^+^CD8^+^4G2^+^	CD3^−^CD19^+^4G2^+^	CD14^+^4G2^+^
D1	29.5	26.9	17.0	68.1
D3	47.3	40.6	47.3	zero
D4	0.6	zero	50.7	ND^b^
D5	1.4	1.1	4.0	ND^b^
D7	0.6	zero	zero	zero
D9	zero	zero	zero	3.9
D14	zero	zero	zero	0.7
D15	zero	zero	1.2	zero
D16	zero	1.7	zero	zero

^a^Infection rate was calculated by subtracting the percentage of 4G2^+^ cells in PBMCs who received ZIKV as compared with the background 4G2 labeling profile in PBMCs from the same donor, but that were not exposed to ZIKV (MOCK). Donors D2, D6, D8, D10, D11, D12 and D13 cells were all negative for 4G2 staining. ^b^ND = not determined, as very low amounts of these subpopulations were detected

Analysis of viral envelope protein among distinct PBMC subpopulations revealed that 31.25% (5/16) of donors presented flavivirus protein in CD3^+^CD4^+^ cells, 25% (4/16) in CD3^+^CD8^+^ cells, 31.25% (5/16) in CD3^−^CD19^+^ cells and 18,75% (3/16) in CD14^+^ cells. We also noticed that one donor (D1) presented the viral protein in all subpopulations analyzed, whereas others were positive in one (D7, D9, D14, D15 and D16), two (D4) or three subpopulations (D3 and D5). These results are summarized in Table 2.

In addition, we observed variable percentages of cells in which the viral protein was clearly detected. As shown in Table 2, cells from donor 1 (D1) presented a high percentage of the viral protein in CD3^+^CD4^+^, CD3^+^CD8^+^, CD3^−^CD19^+^ and CD14^+^ subpopulations, whereas cells from donor 3 (D3) presented envelope protein in CD3^+^CD4^+^, CD3^+^CD8^+^ and CD3^−^CD19^+^ subpopulations, while donor 4 (D4) exhibited high percentages of 4G2^+^ cells only in the CD3^−^CD19^+^ subpopulation. In contrast, the percentages of 4G2^+^ cells in the other donors were very low.

In general, MFI levels for 4G2 labeling also increased in infected donors and this augmentation was proportional to the increase in the percentages of 4G2^+^ cells. Samples presenting high relative numbers of 4G2^+^ cells also presented higher MFI. This finding can be observed in Table 3.

**Table 3:** Flow cytometry-based detection (MFI) of flavivirus envelope protein in subpopulations of human peripheral blood mononuclear cells after ex-vivo infection^a^

Donors	PBMC subpopulations (MFI)			
	CD3^+^CD4^+^4G2^+^	CD3^+^CD8^+^4G2^+^	CD3^−^CD19^+^4G2^+^	CD14^+^4G2^+^
D1	201.0	194.00	189.0	266.0
D3	142.0	135.00	167.0	zero
D4	2.0	zero	374.0	ND^b^
D5	27.0	12.00	42.0	ND^b^
D7	19.0	zero	zero	zero
D9	zero	zero	zero	123.0
D14	zero	zero	zero	6.0
D15	zero	zero	27.0	zero
D16	zero	44.00	zero	zero

^a^MFI (Median of fluorescence intensity) was calculated by subtracting the median of 4G2 labeling in PBMCs subpopulations who received ZIKV as compared with the background 4G2 labeling profile in PBMCs subpopulations from the same donor, but that were not exposed to ZIKV (MOCK). Donors D2, D6, D8, D10, D11, D12 and D13 cells were all negative for 4G2 staining. ^b^ND = not determined, as very low amounts of these subpopulations were detected

## Discussion

In the present work, we show that PBMCs derived from healthy donors can be infected ex vivo by the ZIKV. Using the 4G2 monoclonal antibody, we detected, by flow cytometry, flavivirus envelope protein in 56.25% of PBMCs from healthy donors. In contrast, by RT-qPCR, we detected ZIKV viral loads in supernatant of all infected PBMCs culture, indicating a 100% of infection among tested donors. These data suggest that flow cytometry may not be the most sensitive or reliable method for detecting ZIKV infection in peripheral cells of the immune system, in comparison with the employed viral RNA detection assay.

Overall, our findings are in keeping with recent data showing ex-vivo ZIKV infection in PBMCs from healthy subjects, as well as in cases of in vivo infection by the virus [16, 17]. Michlmayr and colleagues analyzed PBMC susceptibility to ZIKV infection using a strain isolated in Nicaragua (Nica 2–16) and detected the infection mostly in CD14^+^CD16^+^ monocytes and in myeloid dendritic cells. ZIKV infection in NK and B cells was less frequent. These data support the notion that PBMC subpopulations other than monocytes and B and T lymphocytes may be infected by ZIKV. By contrast, Foo and colleagues analyzed PBMC susceptibility to ZIKV infection mediated by two African ZIKV strains, one isolated in Uganda (MR766) and the other in Nigeria (IbH30656), and two Asian ZIKV strains, isolated in Porto Rico (PRVABC59) and in French Polynesia (H/PH/2013), respectively. After whole blood infection with MR766 and H/PH/2013 isolates, ZIKV infection was observed in CD14^+^ monocytes. Direct infection of isolated monocytes showed that the MR766 isolate infects preferentially CD14^high^CD16^+^ monocytes, whereas the H/PH/2013 isolates mainly infect CD14^low^CD16^+^ monocytes. Furthermore, infection of whole blood of healthy pregnant women with MR766 and H/PH/2013 revealed that CD14^+^ monocytes are the primary PBMC target also during pregnancy and that MR766 isolate induced a more robust infection in these cells than the H/PH/2013 isolate. In addition, higher ZIKV loads were detected with both isolates when cells were obtained from women in the first trimester of pregnancy [18]. Using the ZIKV strain isolated in Brazil (RIO-U1), we showed that different PBMC subpopulations are ZIKV targets. Among infected donors, we observed ZIKV infection in CD3^+^CD4^+^ T cells, CD3^+^CD8^+^ T Cells, CD3^−^CD19^+^ B cells and CD14^+^ monocytes, albeit the relative numbers of infected subpopulations varied among donors. In our experiments, we found that the most frequently infected subpopulations were CD3^+^CD4^+^ T cells, CD3^−^CD19^+^ B cells (31.25% of donors tested), followed by CD3^+^CD8^+^ cells (25% of donors) and CD14^+^ monocytes (18,25% of donors). Therefore, our data suggest that, in respect to the RIO-U1 ZIKV isolate, CD3^+^CD4^+^ T cells and CD3^−^CD19^+^ B cells are main ZIKV targets in peripheral mononuclear cells, whereas the CD14^+^ monocytes present the lower permissiveness to RIO-U1 ZIKV isolate.

It is also important to highlight that in previous studies the percentages of PBMCs infected by ZIKV were very low and did not exceed 10% of all viable PBMCs [17, 18]. In contrast, we detected, among cells from different donors, high variability in the numbers of cells infected, reaching up to 68%, as well as in the type of immune cells infected (Table 2).

## Conclusions

In summary, the data presented here indicate that different PBMC subpopulations are targets for ZIKV and that, as least regarding the Brazilian ZIKV isolate applied, several PBMC subsets, including CD4 and CD8 T cells, B cells and monocytes, were infected. These subsets were even able to induce the production of different amounts of viral titers. Infection of these cells by ZIKV could not only facilitates virus dissemination, but also enable virus entry in immune-privileged sites, since these cells are able to cross, for example, the blood-brain barrier, thus playing important role in the pathophysiology of the disease.

## Supplementary information

**Additional file 1:** **Figure S1.** Gate strategy for detection of ZIKV protein in PBMC subpopulations by flow cytometry. PBMCs were analyzed by flow cytometry 72 h post infection at MOI 0.1. We first gated in the lymphocyte and in the monocyte regions based on the forward scattering (FSC, defining size) and side scattering (SCC, defining granularity) profiles. In the lymphocyte gate we analyzed CD3^+^ cells (T lymphocytes) and CD3^−^CD19^+^ cells (B lymphocytes). Within the B lymphocyte subpopulation we evaluated the presence of CD19^+^4G2^+^ (B lymphocytes positive for the ZIKV protein). In respect to T lymphocytes, we further gated in CD4^+^ and in CD8^+^ subpopulations, obtaining CD3^+^CD4^+^ and CD3^+^CD8^+^ T cell subsets, respectively. In each of these subsets we verified the percentages of CD3^+^CD4^+^4G2^+^ and CD3^+^CD8^+^4G2^+^ cells. In the region of monocytes (defined by the forward versus side scattering profiles), we did a gate in CD11c^+^CD14^+^ (monocytes) and inside this subpopulation we verified the percentage of CD14^+^4G2^+^ cells (monocytes positive for ZIKV protein).

**Additional file 2: Figure S2.** ZIKV viral multiplication in PBMCs. (A) PBMCs were infected by ZIKV at MOI 0.1 for 2 h. Supernatant of MOCK, inactivated ZIKV or ZIKV infected PBMC cultures were harvested 24, 48 and 72 h post infection (hpi) and serial dilutions of the supernatant were used to infect Vero cell monolayers. Results are shown as plaque-forming units per mL. Each graph represents one donor (*n* = 2). (B) Histogram analysis of MOCK, inactivated ZIKV and ZIKV infected Vero cells by flow cytometry (*n* = 1).

## Data Availability

Data analyzed during the current study are available from the corresponding author on reasonable request.

## Abbreviations

CHIKV: Chikungunya virus
DENV: Dengue virus
PBMC: Peripheral blood mononuclear cells
RT-qPCR: Quantitative real time reverse transcription polymerase chain reaction
ZIKV: Zika virus
