# Supplementary figures and images for: Zika virus infects human blood mononuclear cells
Source: BMC Infect Dis. 2019 Nov 21;19:986. doi: 10.1186/s12879-019-4622-y (PMC6873492; doi:10.1186/s12879-019-4622-y)

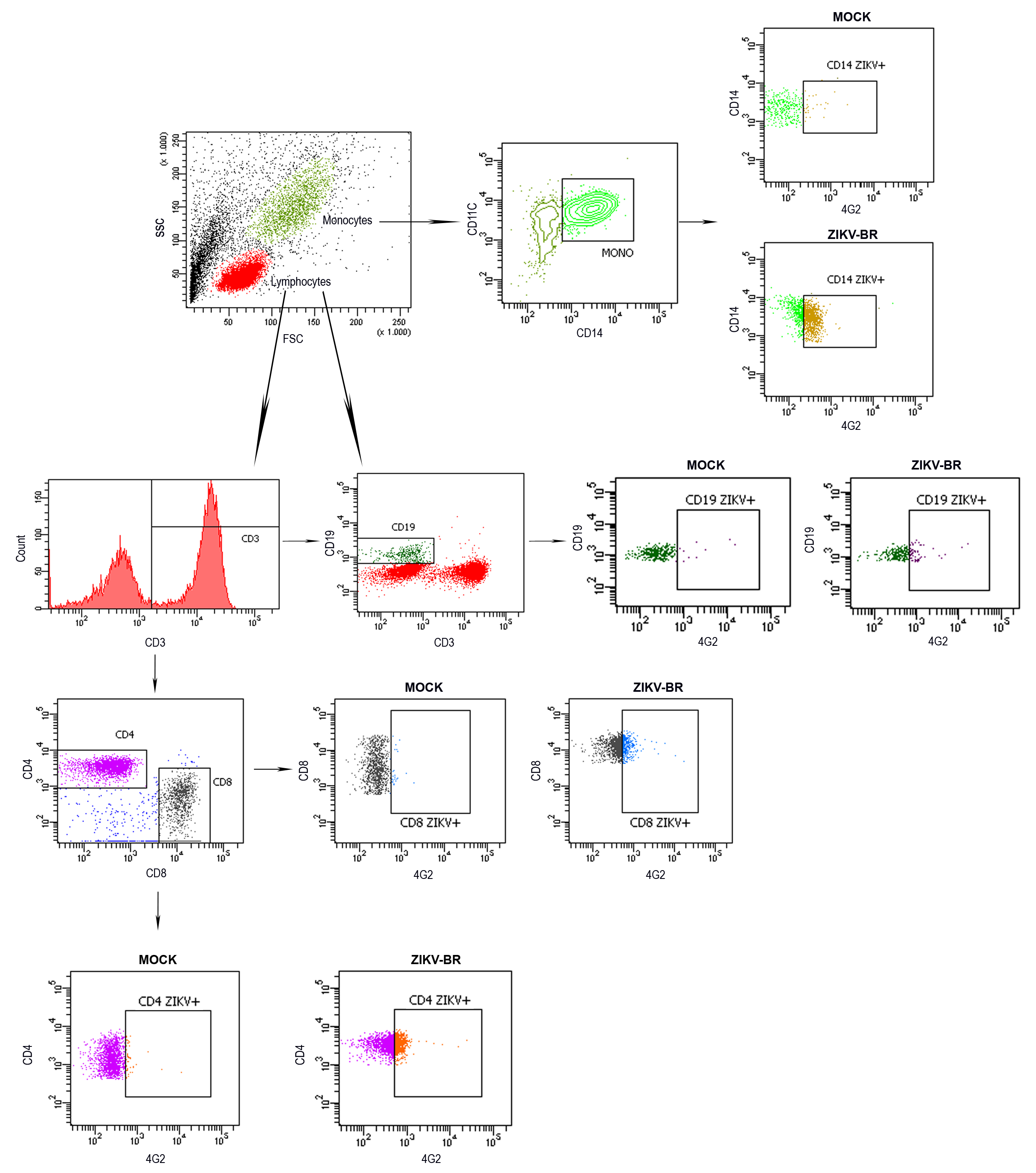

Supplement: Supplementary file 1 — Additional file 1: Figure S1. Gate strategy for detection of ZIKV protein in PBMC subpopulations by flow cytometry. PBMCs were analyzed by flow cytometry 72 h post infection at MOI 0.1. We first gated in the lymphocyte and in the monocyte regions based on the forward scattering (FSC, defining size) and side scattering (SCC, defining granularity) profiles. In the lymphocyte gate we analyzed CD3+ cells (T lymphocytes) and CD3−CD19+ cells (B lymphocytes). Within the B lymphocyte subpopulation we evaluated the presence of CD19+4G2+ (B lymphocytes positive for the ZIKV protein). In respect to T lymphocytes, we further gated in CD4+ and in CD8+ subpopulations, obtaining CD3+CD4+ and CD3+CD8+ T cell subsets, respectively. In each of these subsets we verified the percentages of CD3+CD4+4G2+ and CD3+CD8+4G2+ cells. In the region of monocytes (defined by the forward versus side scattering profiles), we did a gate in CD11c+CD14+ (monocytes) and inside this subpopulation we verified the percentage of CD14+4G2+ cells (monocytes positive for ZIKV protein). [file 12879_2019_4622_MOESM1_ESM.tif]

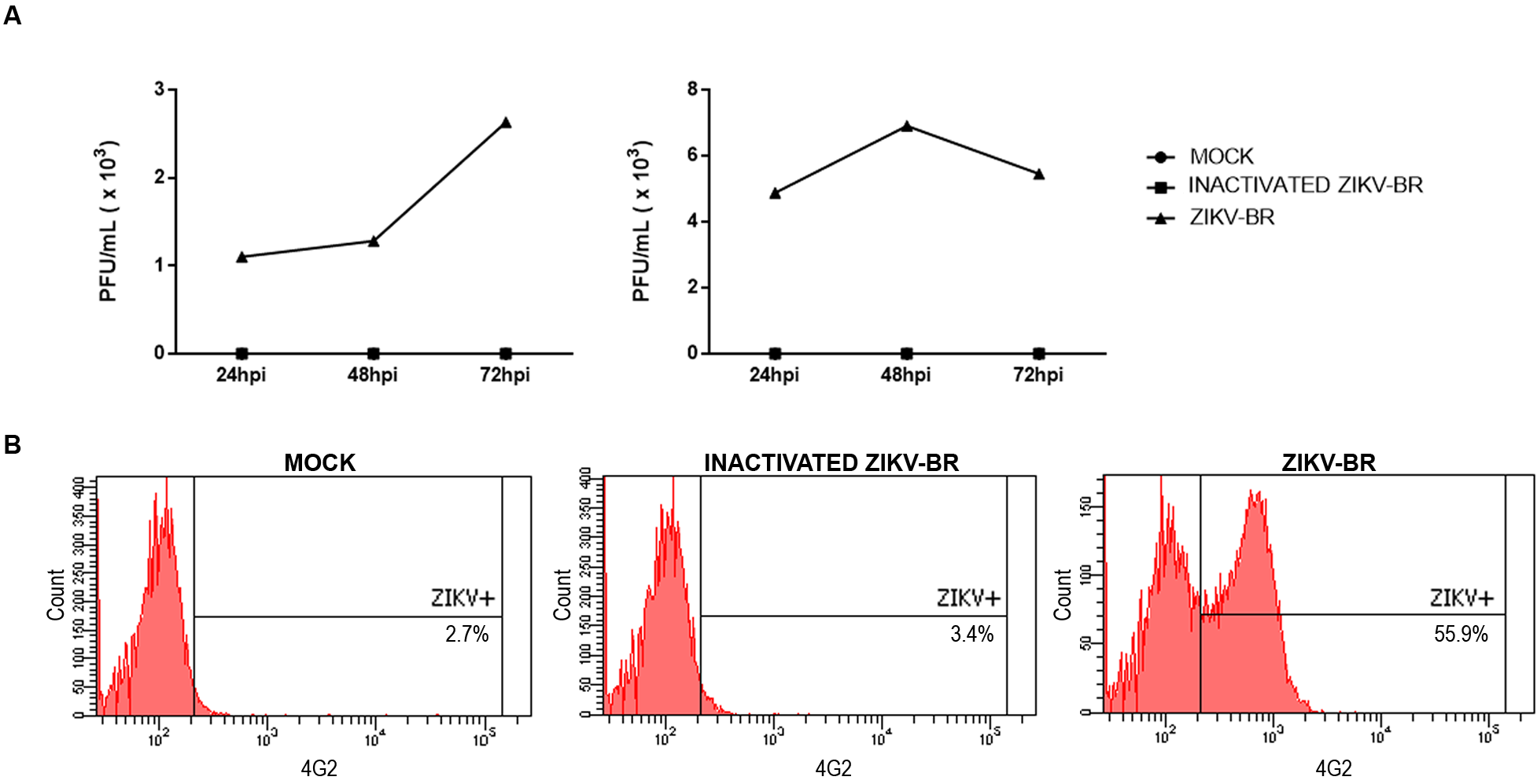

Supplement: Supplementary file 2 — Additional file 2: Figure S2. ZIKV viral multiplication in PBMCs. (A) PBMCs were infected by ZIKV at MOI 0.1 for 2 h. Supernatant of MOCK, inactivated ZIKV or ZIKV infected PBMC cultures were harvested 24, 48 and 72 h post infection (hpi) and serial dilutions of the supernatant were used to infect Vero cell monolayers. Results are shown as plaque-forming units per mL. Each graph represents one donor (n = 2). (B) Histogram analysis of MOCK, inactivated ZIKV and ZIKV infected Vero cells by flow cytometry (n = 1). [file 12879_2019_4622_MOESM2_ESM.tif]
